# Activity of Protease-Activated Receptors in Primary Cultured Human Myenteric Neurons

**DOI:** 10.3389/fnins.2012.00133

**Published:** 2012-09-12

**Authors:** Eva M. Kugler, Gemma Mazzuoli, Ihsan E. Demir, Güralp O. Ceyhan, Florian Zeller, Michael Schemann

**Affiliations:** ^1^Human Biology, Technische Universität MünchenFreising, Germany; ^2^Department of Surgery, Klinikum Rechts der Isar, Technische Universität MünchenMunich, Germany; ^3^Department of Surgery, Klinikum FreisingFreising, Germany

**Keywords:** primary culture, myenteric neurons, protease-activated receptor, thrombin, enteric nervous system

## Abstract

Activity of the four known protease-activated receptors (PARs) has been well studied in rodent enteric nervous system and results in animal models established an important role for neuronal PAR2. We recently demonstrated that, unlike in rodents, PAR1 is the dominant neuronal protease receptor in the human submucous plexus. With this study we investigated whether this also applies to the human myenteric plexus. We used voltage sensitive dye recordings to detect action potential discharge in primary cultures of human myenteric neurons in response to PAR activating peptides (APs). Application of the PAR1-AP (TFLLR) or PAR4-AP (GYPGQV) evoked spike discharge in 79 or 23% of myenteric neurons, respectively. The PAR1-AP response was mimicked by the endogenous PAR1 activator thrombin and blocked by the PAR1 antagonists SCH79797. Human myenteric neurons did not respond to PAR2-AP. This was not due to culture conditions because all three PAR-APs evoked action potentials in cultured guinea pig myenteric neurons. Consecutive application of PAR-APs revealed coexpression (relative to the population responding to PAR-APs) of PAR1/PAR2 in 51%, PAR1/PAR4 in 43%, and of PAR2/PAR4 in 29% of guinea pig myenteric neurons. Our study provided further evidence for the prominent role of neuronal PAR1 in the human enteric nervous system.

## Introduction

Proteases are important for digestive degradation but are at the same time signaling molecules in the gut (Vergnolle, 2005; Hollenberg, 2010). They initiate cellular responses through interactions with four G-protein coupled protease-activated receptors (PARs; PAR1-4; Vu et al., 1991; Nystedt et al., 1995; Ishihara et al., 1997; Kahn et al., 1998; Xu et al., 1998). PARs are activated by specific proteolytic cleavage of their amino terminal extracellular domain which then functions as a tethered ligand (Vergnolle, 2005). Endogenous PAR activators are serine proteases such as thrombin which mainly activates PAR1 or the mast cell derived tryptase which acts as a PAR2 activator. Actions of endogenous proteases are mimicked by synthetic PAR activating peptides (PAR-APs) which contain the amino acid sequence of the respective tethered ligand. PAR-APs are available for PAR1, PAR2, and PAR4 (Corvera et al., 1999; Linden et al., 2001).

PARs are expressed on various cell types in the gut including endothelial cells, epithelial cells, smooth muscle cells, immune cells, and enteric neurons – both in the submucous and myenteric plexus (Al Ani et al., 1995; Mari et al., 1996; Corvera et al., 1999; Buresi et al., 2001; Cenac et al., 2002; Gao et al., 2002; Garrido et al., 2002; Mall et al., 2002; Colognato et al., 2003; Kawabata et al., 2004; Vergnolle, 2005; Li et al., 2008). Activation of PARs cause activation of rodent enteric neurons as indicated by increased spike discharge or increase in intracellular calcium level (Corvera et al., 1999; Linden et al., 2001; Gao et al., 2002; Reed et al., 2003). More than 50% of rodent myenteric neurons responded to PAR1 or PAR2 activators but only 25% to PAR4 activators (Corvera et al., 1999; Linden et al., 2001; Gao et al., 2002).

There is strong evidence for involvement of PARs in functional and inflammatory bowel diseases. In the stool of patients with ulcerative colitis (UC) and irritable bowel syndrome (IBS) increased concentrations of proteases were found (Bustos et al., 1998; Cenac et al., 2007; Roka et al., 2007; Annahazi et al., 2009). Additionally, mucosal biopsy supernatants from IBS patients contained higher protease levels than supernatants from healthy controls (Barbara et al., 2004; Buhner et al., 2009). One functional consequence of the enhanced protease release has been demonstrated in sensory and enteric neurons. Thus, supernatants taken from colonic biopsy samples of IBS patients sensitized murine sensory neurons and induced visceral and somatic hypersensitivity through activation of PAR2 (Barbara et al., 2007; Cenac et al., 2007; Buhner et al., 2009). In addition, IBS supernatants evoked spike discharge in human submucous neurons (Barbara et al., 2007; Buhner et al., 2009). In both studies the unspecific serine protease inhibitor FUT-175 inhibited neuronal responsiveness to IBS supernatants.

Recently, we demonstrated that PAR1 rather than PAR2 or PAR4 activators excited human submucous neurons (Mueller et al., 2011). In this study we furthermore confirmed that PAR2 was indeed most prominent in the guinea pig submucous plexus. Although the results suggested important species differences and plexus differences, it remained open whether the differential functional PAR expression is also relevant in human myenteric neurons. We therefore studied actions of PAR1, PAR2, and PAR4 activators in cultured human myenteric neurons and performed comparative studies in cultured guinea pig myenteric neurons. We have recently reported that recordings from cultured human myenteric neurons more reliably reveal representative results than from freshly dissected preparations (Vignali et al., 2010). Furthermore we analyzed the PAR clustering in cultured guinea pig myenteric neurons by pairwise application of PAR-APs.

## Materials and Methods

### Primary culture of human and guinea pig myenteric neurons

Human tissue samples from small (5) and large (21) intestine were obtained from 26 patients [16 female and 10 male; 69.7 ± 2.2 years (mean ± SEM)] undergoing surgery at the Medical Clinics in Freising and Rechts der Isar in Munich. Samples were taken from macroscopically unaffected areas. Diagnoses that led to surgery were carcinoma (23), polyp (1), ileus (1), and diverticulitis (1). Procedures were approved by the ethical committee of the Technische Universität München (1746/07; informed consent was obtained from all subjects). After surgery, tissue samples were placed in ice-cold oxygenated sterile Krebs solution and immediately transferred to our institute. Tissues were dissected in ice-cold oxygenated sterile Krebs solution containing (in mM): 121 NaCl, 6 KCl, 12 Glucose, 14 NaHCO_3_, 1 NaH_2_PO_4_, 1 MgCl_2_·6H_2_O, 3 CaCl_2_·2H_2_O. These tissues were dissected in order to obtain longitudinal muscle – myenteric plexus (LMMP) preparations. Culture procedures were performed after a previously described method to gain primary culture of human myenteric neurons (Vignali et al., 2010). Similar preparations were done with guinea pig intestinal tissues. Briefly, male guinea pigs (Dunkin Hartley, Harlan GmbH, Borchen, Germany) were killed by cervical dislocation followed by exsanguination (approved by the local animal ethical committee and according to the German guidelines for animal protection and animal welfare). A 10 cm piece of the distal small intestine was quickly removed. Further proceedings in human and guinea pig were performed under sterile conditions. The sterile LMMP preparations of both species were cut in ∼1 mm × 1 mm pieces and digested for 20–70 min in an enzymatic solution containing 0.9 mg/mL protease type I from bovine pancreas (Sigma-Aldrich, Steinheim, Germany), 1.2 mg/mL collagenase type II from *Clostridium histolyticum* (Gibco, Karlsruhe, Germany) and 3.7 mg/mL bovine serum albumin fraction V (Serva, Heidelberg, Germany). Enzymatic digestion was followed by washing steps with ice-cold sterile Krebs solution. The obtained pellet was dissolved in medium M199 (Gibco). From this suspension ganglia were picked under a stereomicroscope (Leica DMIL, 4× objectives with phase contrast) and 2–5 dishes (Ibidi, Martinsried, Germany) were inoculated with ganglia suspension. Myenteric ganglia were incubated in medium M199 supplemented with 10% FBS (Gibco), 50–100 ng/mL mouse nerve growth factor 7S (Alomone labs, Jerusalem, Israel), 5 mg/mL Glucose, 100 U/mL Penicillin, 100 μg/mL Streptomycin (Gibco), and 2 μM arabinose-C-furanoside (Sigma-Aldrich). The medium was changed every 2–3 days. At the first day after the culture the enteric neurons adhere to the bottom of the dish. After 48–72 h the neurons tend to reorganize themselves in structures defined as cluster that form a network. The cultures were grown at least for 7 days before performing an experiment.

For voltage sensitive dye recordings the dishes were placed in a custom made recording chamber and continuously perfused with 37°C HEPES solution containing (in mM): 1 MgCl_2_·6H_2_O, 2 CaCl_2_·2H_2_O, 150 NaCl, 5 KCl, 10 Glucose, 10 HEPES.

### Multisite optical recording technique with voltage sensitive dye

An ultrafast neuroimaging technique was used to record signals from the fluorescent potentiometric dye 1-(3-sulfanato-propyl)-4-[β-[2-(di-n-octylamino)-6-naphthyl] vinyl] pyridinium betaine (Di-8-ANEPPS; Invitrogen, Darmstadt, Germany) as previously described in detail (Neunlist et al., 1999; Schemann et al., 2002; Michel et al., 2011). The cultured neurons were stained by incubation with 500 μL of 10 μM Di-8-ANEPPS for 15 min at room temperature in the dark.

The recording chamber was mounted onto an inverted epifluorescence microscope (Zeiss Axio Observer.A1; Munich, Germany). To detect the signals of Di-8-ANEPPS the microscope was equipped with a modified Cy3 filterset (545 ± 15 nm excitation, 565 nm dichroic mirror, 580 nm barrier; Ahf Analysentechnik, Tübingen, Germany) and excited by a green LED (LE T S2W, Osram, Munich, Germany). We used the Neuroplex 9.1.1 software (RedShirtImaging, Decatur, GA, USA) for acquisition and processing of the signals. The changes of the membrane potential are linearly related to the relative changes of the fluorescence (ΔF/F) which was measured by a cooled charge coupled device (CCD) camera (80 × 80 pixels, RedShirtImaging). Optical signals were recorded with a frame rate of 1 kHz which enables the detection of action potentials. With an x100 objective (NA = 1.35, Olympus, Hamburg, Germany) the spatial resolution of the CCD camera is ∼4.0 μm^2^ per pixel. The visualization of the outlines of the cells marked by the dye that is incorporated in the cell membrane make it possible to detect the signal of each single cell (Michel et al., 2005).

### Drugs and solutions

Amino acid sequences that specifically activate the different PAR receptors were used as PAR-APs. We used human specific PAR-APs (PAR1-AP: TFLLR-NH_2_; PAR2-AP: SLIGKV-NH_2_; PAR4-AP: GYPGQV-NH_2_) or rodent specific PAR-APs (PAR1-AP: SFFLR-NH_2_; PAR2-AP: SLIGRL-NH_2_; PAR4-AP: GYPGKF-NH_2_). For control experiments we used in human myenteric neurons also the reversed peptide (RP) of PAR1 (PAR1-RP: RLLFT-NH_2_; all from Peptide Synthesis Core Facility, University of Calgary, Calgary, Alberta, Canada). 10 mM stock solutions of PAR-APs and PAR1-RP were prepared with deionized water and stored at −20°C. Before experiments they were further diluted with HEPES solution to 100 μM. With human plasma derived thrombin (citrate-free; Merck KGaA, Darmstadt, Germany) a stock solution at a concentration of 57.83 μM diluted in deionized water was prepared and stored at −80°C. Final concentration of 100 nM was obtained after dilution with HEPES solution. The PAR1 antagonist SCH79797 (N^3^-cyclopropyl-7-[[4-(1-methylethyl)phenyl] methyl]-7H-pyrrolo[3,2-f]quinazoline-1,3-diamine dihydrochloride; Biozol Diagnostica, Eching, Germany) was dissolved in waterfree 100% dimethyl sulfoxide (Acros Organics, Geel, Belgium; Ahn et al., 2000). For experiments SCH79797 was added at a final concentration of 5 μM for 20 min to the superfusing HEPES solution. For all substances the pH was 7.4. To identify neurons 100 μM nicotine (Sigma-Aldrich) was applied by pressure ejection from a glass pipette positioned about 500 μm away from the cell cluster. PAR1-AP, PAR2-AP, PAR4-AP, PAR1-RP were also applied by pressure ejection at a concentration of 100 μM, thrombin at a concentration of 100 nM. These substances are diluted during pressure ejection by a factor of 1:5–1:10 before reaching the cluster (Breunig et al., 2007). PAR-APs as well as thrombin evoke a late onset neuronal response (Mueller et al., 2011). We therefore adapted the microejection protocol as follows: we applied the PAR activators for 2 s and recorded the response of the neurons in four 2 s long trials; which were separated by 5–6 s intervals without recording. For analysis of PAR clustering PAR-APs were applied pairwise in random order.

### Data analysis and statistics

The number of neurons responding to nicotine in the field of view was counted and based on this number the percentage of neurons responding to PAR activators per cluster was analyzed as well as the spike discharge frequency. With the mean spike discharge of the responding neurons per cluster multiplied with the percentage of responding neurons in this cluster the neuroindex was calculated and used for illustration of the results. As mentioned above, PAR activators evoke a late onset spike discharge (Mueller et al., 2011). We therefore analyzed spike discharge frequency during the last three trials as there were rarely spikes during the first acquisition. PARs desensitize to repeated application of activators (Gao et al., 2002). We therefore studied blockade of PAR1 by SCH79797 by comparing PAR1-AP effects before and during perfusion of the PAR1 antagonist in two different clusters obtained from the same patient. We analyzed the voltage sensitive dye signals with Neuroplex 8.3.2 (RedShirtImaging), Igor Pro 6.12A (Wavemetrics Inc., Lake Oswego, OR, USA) and SigmaPlot 12 (Systat Software Inc., Erkrath, Germany). Since all data were not normally distributed they are presented in the text as median with their 25 and 75% quartiles. Differences of responses were tested with Rank Sum Test. Multiple comparisons were performed with Kruskal–Wallis one-way analysis of variance (ANOVA) on ranks. Differences were considered significant when *p* < 0.05. For readability the numbers of patients, clusters, and neurons are all presented in the figure legend and only mentioned in the text if not graphically illustrated.

## Results

**Figure 1:**
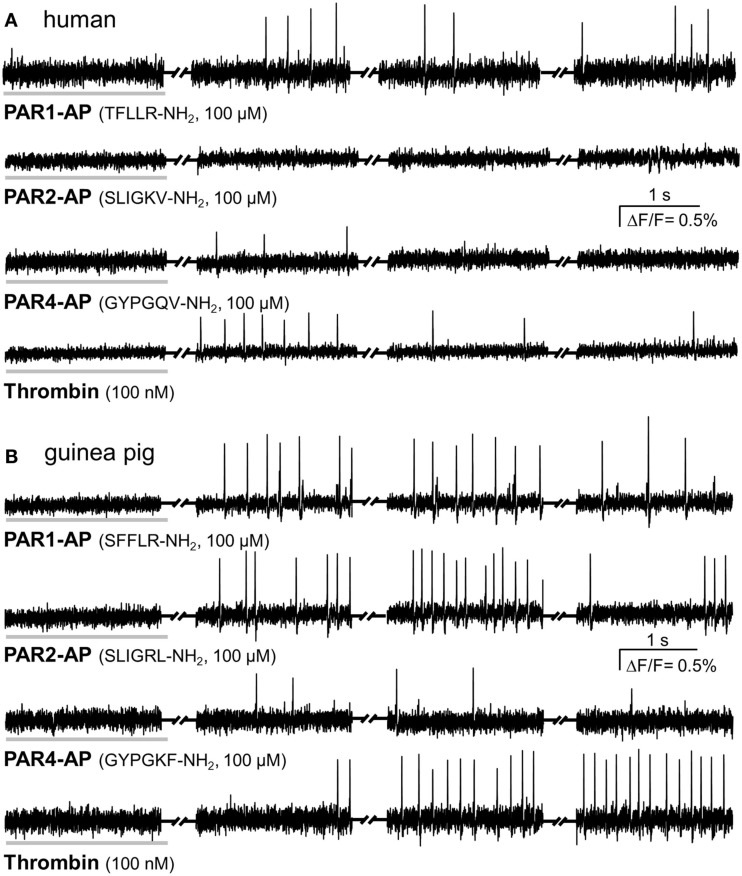
**PAR-APs and thrombin activate cultured myenteric neurons**. Representative traces of voltage sensitive dye recordings showing neuronal responses to a 2 s spritz application (indicated by the horizontal gray bar) of PAR1-AP, PAR2-AP, PAR4-AP, and thrombin. Recordings were made in four 2 s long recording periods with 5–6 s intervals in between (indicated by the symbol between the traces). Every peak represents an action potential. **(A)** Representative traces from cultured human myenteric neurons to human specific PAR-APs and thrombin show comparable responses to PAR1-AP and thrombin but no response to PAR2-AP and a minor response to PAR4-AP. **(B)** Guinea pig cultured myenteric neurons fire action potentials in response to PAR1-AP, PAR2-AP, PAR4-AP, and thrombin; the PAR4 response is rather moderate.

**Figure 2:**
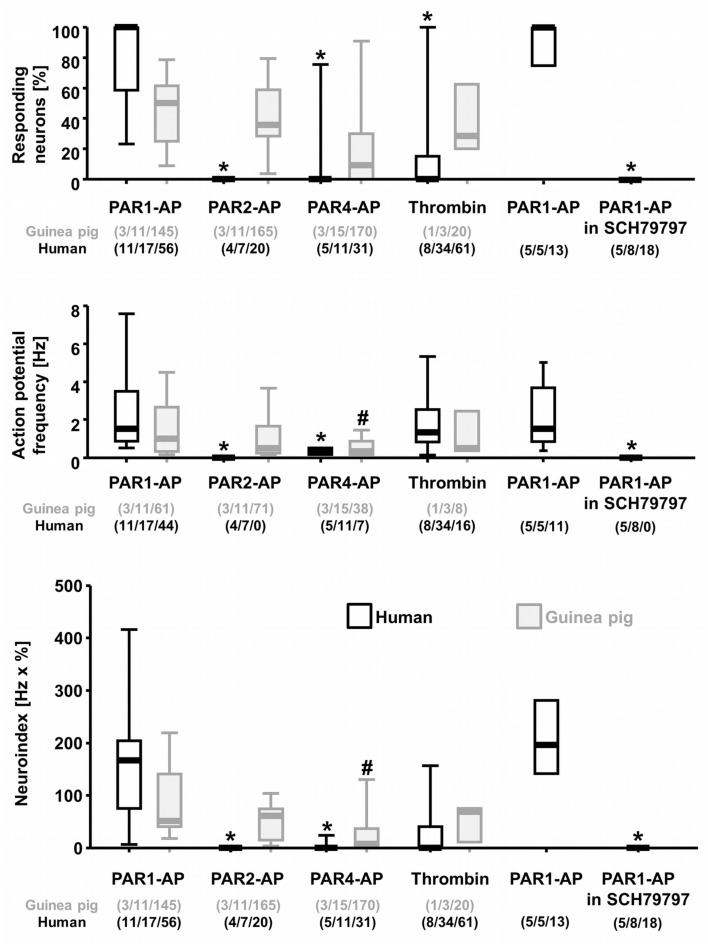
**Analysis of neural actions of PAR-APs and thrombin in cultured human and guinea pig myenteric neurons**. The graphs represent (from top to bottom) the proportion of neurons per cluster responding to a specific PAR activator, the specific PAR activator evoked spike frequency and the neuroindex which is the product of spike frequency and proportion of responding neurons. Data are illustrated with scatter plots showing the 25%/75% and the bars indicating the 10%/90% percentiles. PAR1-AP induced the strongest effect in human myenteric neurons, whereas PAR4-AP evoked only weak responses and PAR2-AP no response. Thrombin also activated human myenteric neurons but to a lesser degree than PAR1-AP. The PAR1-AP effect in human neurons is blocked by the specific PAR1 antagonist SCH79797. In guinea pig myenteric neurons the responsiveness to PAR1-AP, PAR2-AP, and thrombin was similar, but PAR4-AP evoked a spike discharge in less neurons at a significantly lower frequency. *Indicates significant differences to PAR1-AP in human neurons; ^#^indicates significant differences to PAR1-AP in guinea pig neurons. Numbers in parenthesis indicate numbers of tissue/clusters/neurons.

PAR1-AP induced a considerable spike discharge in human myenteric neurons, whereas PAR4-AP or PAR2-AP evoked significantly less or no spike discharge, respectively (Figures 1 and 2). The neuroindex in Figure 2 is a product from the proportion of responding neurons per cluster and the PAR activator evoked mean spike discharge. PAR1-AP application evoked spike discharge in 79% (44 of 56 neurons) of the neurons at a median frequency of 1.5 (0.9/3.5) Hz. PAR2-AP failed to activate any human myenteric neurons (0 of 20 neurons). To PAR4-AP application only 23% (7 of 31 neurons) of myenteric neurons fired at a median frequency of 0.3 (0.2/0.5) Hz. PAR1-AP application activated significantly more neurons and evoked a significantly higher spike discharge (*p* ≤ 0.001; Figure 2).

The response to PAR1-AP was specific as application of PAR1-RP had no effect (seven neurons from four clusters and two patients). In addition, the PAR1 specific antagonist SCH79797 (5 μM) fully abolished the response to PAR1-AP (Figure 2). While control applications of PAR1-AP evoked a response in 85% (11 of 13 neurons) of neurons, application of PAR1-AP in the presence of SCH79797 evoked no action potentials in any neuron (0 of 18 neurons; *p* ≤ 0.001).

In rodent myenteric neurons PAR2 activation evoked a reliable activation (Corvera et al., 1999; Linden et al., 2001; Gao et al., 2002). We wanted to exclude that our culture conditions would negatively influence PAR2 activation. We therefore studied the effects of rodent specific PAR1-AP, PAR2-AP, and PAR4-AP on guinea pig primary cultured myenteric neurons. PAR1-AP activated 42% (61 of 145 neurons) of guinea pig myenteric neurons with a median spike discharge of 1.0 (0.3/2.7) Hz. PAR2-AP evoked a similar activation of neurons and induced a median spike discharge of 0.5 (0.3/1.7) Hz in 43% (71 of 165 neurons) of myenteric neurons. PAR4-AP activated 22% (38 of 170 neurons) of myenteric neurons with a median spike discharge of 0.3 (0.2/0.9) Hz.

**Figure 3:**
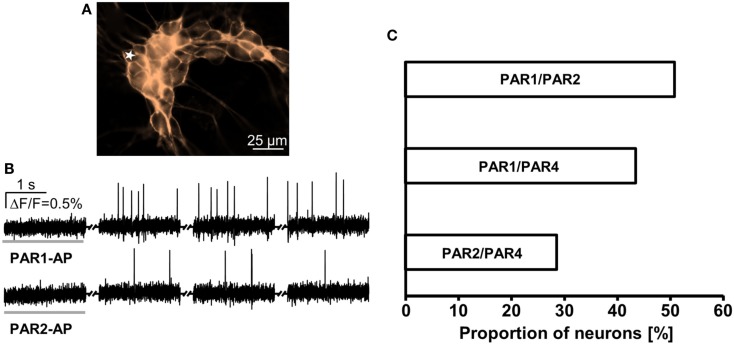
**Response pattern to PAR-APs in guinea pig cultured myenteric neurons demonstrates receptor clustering**. **(A)** Image shows outlines of a cluster of guinea pig cultured myenteric neurons stained with the voltage sensitive dye Di-8-ANEPPS. **(B)** Representative traces of a neuron [marked with a star in **(A)**] that responded to PAR1-AP and PAR2-AP (2 s spritz application indicated by a gray horizontal bar). Recordings were made in four 2 s long recording periods with 5–6 s intervals in between (indicated by the symbol between the traces). Every peak represents an action potential. **(C)** Pairwise application of PAR-APs revealed functional coexpression patterns. Proportions of neurons responding to two PAR-APs are expressed relative to the proportion of neurons responding to any PAR-AP in that particular set of experiment (100%).

In order to determine clustering of PAR in guinea pig myenteric neuron cultures we pairwise applied PAR-APs. Applications of PAR1-AP and PAR2-AP revealed that 51% of the neurons (31 out of 61) responded to both, 34% (21 out of 61) to PAR1-AP only, and 15% (9 out of 61) to PAR2-AP only. Similar experiments with PAR1-AP and PAR4 AP showed that 43% of the neurons (20 out of 46) responded to both, whereas 39% (18 of 46) to PAR1-AP only and 17% (8 out of 46) to PAR4-AP only. Applications of PAR2-AP and PAR4-AP revealed that 29% of neurons (24 out of 84) responded to both, 46% (39 out of 84) to PAR2-AP only, and 25% (21 of 84) to PAR4-AP only (Figure 3).

PAR1-AP by far evoked the strongest activation in human myenteric neurons. The endogenous PAR1 activator thrombin evoked in 26% (16 of 61 neurons) of human myenteric neurons a median spike discharge of 1.3 (0.8/2.5) Hz (Figures 1 and 2). Thus PAR1-AP activated significantly more neurons than thrombin (*p* ≤ 0.001). This was due to the relatively high proportion of clusters in which no neurons responded to thrombin. This was the case in 71% of the clusters; in the remaining 29% thrombin activated as many neurons as the PAR1-AP [56% (28 of 50 neurons) vs. 79% (44 of 56 neurons)]. Thrombin furthermore induced a similar spike discharge as PAR1-AP in these neurons [1.3 (0.8/2.5) vs. 1.5 (0.9/3.5) Hz]. In guinea pig cultured myenteric neurons 40% (8 of 20 neurons) were activated by thrombin with a spike discharge of 0.5 (0.4/2.5) Hz.

## Discussion

The results of our study provided further support for the dominant role of PAR1 in the human enteric nervous system. We found that both the PAR1-AP as well as the endogenous activator thrombin excited human myenteric neurons. In contrast, PAR4-AP evoked less activation while PAR2-AP had no effect. The failure of PAR2-AP to evoke spike discharge was not due to culture conditions as PAR2 activation readily evoked spike discharge in cultured guinea pig myenteric neurons. These findings expand on our previous observation that PAR1 rather than PAR2 activators induce neuronal activation in the human submucous plexus (Mueller et al., 2011). We thereby demonstrated that PAR2 played a minor role in both the submucous and myenteric plexus whereas neuronal PAR1 dominated in both plexi of the human enteric nervous system. These differences between human and rodent enteric neurons must be considered when translating findings on involvement of PARs from animal models to human gut pathology. The physiological relevance of this striking species difference remains to be explored.

In guinea pig cultured myenteric neurons PAR1-AP, PAR2-AP, and thrombin evoked similar responses, whereas less neurons responded to PAR4-AP. This finding agrees with data in non-dispersed (Linden et al., 2001; Gao et al., 2002) and dispersed myenteric plexus neurons (Corvera et al., 1999). We found evidence for a functional PAR clustering in cultured myenteric neurons. Our study revealed that 51% responded to PAR1 and PAR2, a value that is almost identical to the ∼60% reported from a similar study in dispersed myenteric neurons (Corvera et al., 1999). In addition, we provided first data on clustering of PAR1 with PAR4 (43%) and PAR2 with PAR4 (29%).

We found that 79% of human myenteric neurons fired action potentials in response to PAR1-AP. This proportion is very similar to the PAR1-AP responsive neurons in the human submucous plexus (71%, Mueller et al., 2011). In addition the endogenous PAR1 activator thrombin evoked spike discharge in 26% of human myenteric neurons. In our experiments in cultured human myenteric neurons much less neurons responded to thrombin in comparison to the PAR1-AP. In freshly dissected guinea pig preparations the neuronal responsiveness to thrombin and PAR1-AP was quite similar (Corvera et al., 1999; Hollenberg and Compton, 2002). One possible explanation could be differences in potency although thrombin appears to have a higher potency than the PAR1-AP (Corvera et al., 1999; Hollenberg and Compton, 2002). Another reason may be the culture conditions which require the use of proteases for tissue digestion. During the culture period the neurons may be exposed to unspecified proteases present in the supplemented serum. We cannot exclude that some of these proteases may inactivate or disarm PAR1, which has been described for cathepsin G, plasmin, elastase, proteinase-3, and trypsin (Ossovskaya and Bunnett, 2004; Hollenberg et al., 2008; Ramachandran et al., 2012). In the disarmed state the PAR1 can be still activated by the tethered ligand, but not by thrombin. Irrespective of whether culture conditions may affect PAR1 activation by thrombin, our results clearly demonstrated that thrombin activates human myenteric neurons.

Strikingly, while PAR2-AP activated at least a small population of human submucous neurons (6.3%) it did not evoke spikes in any human myenteric neurons. This may be a reflection of the pronounced gradient of mast cells, which release tryptase as the main endogenous PAR2 activator, in the human gut wall (Buhner and Schemann, 2012). While there was a close anatomical association between mast cells and nerves in the submucous layer, there were hardly any mast cells in the human myenteric layer. We may exclude that the dispersal procedures or receptor internalization interfered with PAR2 activation in human cultured neurons for several reasons. Firstly, identical culture conditions did not negatively affect PAR2 activation in guinea pig myenteric neuron culture. Secondly, inability to record PAR responses due to receptor internalization would be expected to affect all PARs; but we readily evoked responses to PAR1-AP and PAR4-AP in cultured human myenteric neurons. We are not aware of data suggesting that inactivation of PAR2 by internalization is more pronounced than for PAR1 or PAR4.

Our results do not rule out involvement of non-neuronal PAR2 in the regulation of gastrointestinal functions in humans. PAR2 are functionally expressed on epithelial cells, myofibroblasts, smooth muscle cells, and PAR2 activation does evoke direct responses in these cells (Seymour et al., 2005; Sato et al., 2006; Kawabata et al., 2008; Tanaka et al., 2008; Mueller et al., 2011).

PAR1 activation modulates motility and visceral sensitivity in several animal models (Kawabata et al., 2008). Whether this also applies to human intestine is not known. However, clinical trials with PAR1 antagonists as anti-platelet medication reported as side effects in a small number of patients nausea, constipation or visceral pain although their incidence was not significantly higher than for the placebo group (O’Donoghue et al., 2011; Shinohara et al., 2012).

In conclusion, our data provide further evidence for the prominent role of neuronal PAR1 in the human enteric nervous system and suggest their prominent involvement in muscle and epithelial functions.

## Conflict of Interest Statement

The authors declare that the research was conducted in the absence of any commercial or financial relationships that could be construed as a potential conflict of interest.
